# Parameter Screening in Microfluidics Based Hydrodynamic Single-Cell Trapping

**DOI:** 10.1155/2014/929163

**Published:** 2014-06-09

**Authors:** B. Deng, X. F. Li, D. Y. Chen, L. D. You, J. B. Wang, J. Chen

**Affiliations:** ^1^State Key Laboratory of Transducer Technology, Institute of Electronics, Chinese Academy of Sciences, Beijing 100190, China; ^2^Institute of Biomaterials and Biomedical Engineering, University of Toronto, Toronto, ON, Canada M5S 3G9

## Abstract

Microfluidic cell-based arraying technology is widely used in the field of single-cell analysis. However, among developed devices, there is a compromise between cellular loading efficiencies and trapped cell densities, which deserves further analysis and optimization. To address this issue, the cell trapping efficiency of a microfluidic device with two parallel micro channels interconnected with cellular trapping sites was studied in this paper. By regulating channel inlet and outlet status, the microfluidic trapping structure can mimic key functioning units of previously reported devices. Numerical simulations were used to model this cellular trapping structure, quantifying the effects of channel on/off status and trapping structure geometries on the cellular trapping efficiency. Furthermore, the microfluidic device was fabricated based on conventional microfabrication and the cellular trapping efficiency was quantified in experiments. Experimental results showed that, besides geometry parameters, cellular travelling velocities and sizes also affected the single-cell trapping efficiency. By fine tuning parameters, more than 95% of trapping sites were taken by individual cells. This study may lay foundation in further studies of single-cell positioning in microfluidics and push forward the study of single-cell analysis.

## 1. Introduction

The goal of current cellular biology studies is to understand the molecular mechanisms underlying cellular functions [1]. Most cell-based assays (e.g., western blot and bulk PCR) collect data averaged across large cell populations and thus overlook rich information available when single cells are studied. Meanwhile, it has been known that individual cells with identical appearances differ in biological properties as cellular heterogeneity. Due to this heterogeneity, much effort has been devoted over the past few years in technical developments to study cells in the single-cell level [2, 3].

Among these developed methods, flow cytometry is the most commonly used method for single-cell analysis, enabling simultaneous multiparametric analysis of the biophysical/biochemical properties of single cells in a high-throughput manner [4]. Although powerful, flow cytometry cannot monitor temporal changes of single cells under stimulation and thus its functionality in understanding cellular molecular mechanisms is limited [5].

Quantitative microscopy enables single-cell monitoring in a time-lapse manner where both biophysical (e.g., cellular morphology) and biochemical information (e.g., calcium concentration) can be obtained [1]. However, in conventional culture flasks, uniform environments of single cells cannot be guaranteed due to nonuniform distributions of biochemical and biophysical cues (e.g., glucose, oxygen, and local fluid flow). Thus, microscopy based single-cell analysis using conventional culture flaks leads to compromised results [5].

Recently, microfluidics is under intensive research, which is the science and technology of manipulation and processing of small amounts of fluids [6, 7]. Since its critical dimension is in the microscale, microfluidics has been used to capture, culture, stimulate, and retrieve single biological cells [8–10]. In the field of single-cell capture, both active and passive trapping principles were proposed to enable large-array single-cell positioning in a uniform environment [11–13].

Among active single-cell trapping schemes, dielectrophoresis is the most commonly used method, which confines cells via their inducible electric dipoles in an electric field gradient, featured with selective cellular capture and release [14–16]. However, dielectrophoresis requests fine tuning of the applied electrical parameters, which may lead to trapping of multiple cells due to inappropriate control of the dielectrophoretic forces. In addition, the trapping method is not suitable for long-term cell culture due to potentially cytotoxic low-conductivity buffers and/or high temperatures induced by Joule heating [17].

As to passive methodologies for single-cell trapping, flow-based single-cell positioning has been developed, which can be divided into two categories. In the first strategy, single cells are loaded into individual wells patterned on silicon or polymeric materials due to gravity where the single-cell trapping efficiency is dominated by geometrical parameters of trapping wells and cellular diameters [18, 19]. Although this trapping method is featured with simplicity, its nature of static cell culture limits its possibilities to actively manipulate the trapped cells and conduct temporal stimuli.

In the second strategy, arrays of weirs or dams are used to trap cells as they move through a fluidic device under hydrodynamic forces. Di Carlo et al. firstly presented a hydrodynamic trapping array with U-shaped barriers, where the trapping of one single cell increases the flow resistance significantly and thus following cells travel around the trapping spot [20, 21]. Furthermore, Takeuchi et al. proposed a serpentine design in which single cells were trapped in the trapping sites in sequence [22, 23], which was then scaled up in the design proposed by Lu et al. [24]. Although lots of efforts were devoted in this area, further parameter investigation and optimization on cellular trapping efficiencies are still requested [25].

**Figure 1:**
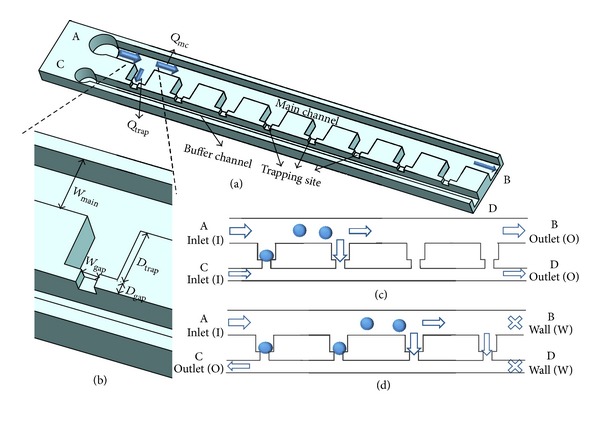
Principle of single-cell positioning based on the hydrodynamic trapping mechanism. (a) Schematic of the cell trapping structure, which consists of two parallel channels (main channel and buffer channel) connected with cellular trapping sites with a decrease in the cross-sectional area. A, B, C, and D represent the main channel inlet, main channel outlet, buffer channel inlet, and buffer channel outlet, respectively. Four critical geometry parameters of the trapping site *W*_main_, *W*_gap⁡_, *D*_trap_, and *D*_gap⁡_ were illustrated in (b). For each channel inlet or outlet, there are three possible statuses, which are I (inlet with a defined fluid flow rate as boundary condition), O (outlet with zero pressure as boundary condition), and W (wall with zero fluid flow velocity as boundary condition). By regulating the status of channel inlets and outlets, this structure can represent previous designs where (c) represents the structure proposed by Shoji et al. [26] and (d) presents the structure put forward by Lu et al. [24].

To address this issue, in this study, we proposed a microfluidic device including two parallel microchannels (main channel and buffer channel) interconnected with cellular trapping sites with a decrease in the cross-sectional area (see Figure 1). By choosing the on/off status of the inlets and outlets of these two channels, this design can represent previous trapping structures where Figure 1(c) represents the structure proposed by Shoji et al. [26, 27] and Figure 1(d) presents the trapping structure put forward by Lu et al. [24]. Numerical simulations were conducted to evaluate the cellular trapping efficiencies for a variety combination of inlets and outlets as well as geometry parameters. In addition, experimental results were conducted to further screen parameters including cellular fluid velocities and sizes for high-efficiency cellular positioning.

## 2. Materials and Methods

### 2.1. Materials and Cell Culture

Unless otherwise indicated, all cell-culture reagents were purchased from Life Technologies Corporation (Van Allen Way Carlsbad, CA, USA). Materials required for device fabrication included SU-8 photoresist (MicroChem Corp., Newton, MA, USA) and 184 silicone elastomer (Dow Corning Corporation, Midland, MI, USA).

A non-small-cell lung cancer cell line A549 was cultured at 37°C in 5% CO_2_ in RPMI 1640 medium supplemented with 10% heat-inactivated fetal bovine serum, 100 units/mL penicillin, and 100 *μ*g/mL streptomycin.

### 2.2. Numerical Simulation

Extensive simulations were conducted using the finite element analysis package COMSOL 4.3 (Burlington, MA, USA) to quantify the effects of channel inlet/outlet status and geometry parameters on the cellular trapping efficiency. In this study, the incompressible Navier-Stokes module (element type: tetrahedral element and element number: 6787192) was used to simulate fluid flow velocity distributions in the microfluidic device. The trapping site flow ratio *Q*_trap_/*Q*_mc_ was defined as the ratio of the fluid volume flow rate through the trapping site and the volume flow rate at the main channel after the cellular trapping site. This parameter was used in previous studies to indicate the possibility of cellular trapping and it was speculated that *Q*_trap_/*Q*_mc_ should be higher than 1, which may enable sequential trapping of single cells [22, 25].

In this simulation, the effect of inlet and outlet status on *Q*_trap_/*Q*_mc_ was investigated. There are 6 status combinations for ports A, B, C, and D under study which are IOOO, IOWO, IWOO, IOOW, IWOW, and IWWO. Note that “I” represents the channel inlet with a defined fluid flow rate, “O” represents the channel outlet with a zero pressure, and “*W*” represents the channel outlet with a zero flow rate. Four key geometry parameters (*W*_gap⁡_, *D*_gap⁡_, *D*_trap_, and *W*_main_, see Figure 1(b)) with adjustable valuables were listed in Table 1 and their effects on *Q*_trap_/*Q*_mc_ were quantified using numerical simulations.

### 2.3. Device Fabrication

The PDMS device with two different channel heights was fabricated based on conventional microfabrication techniques, including two-layer SU-8 mold fabrication, PDMS molding, and sealing with glass substrates.

**Figure 2:**
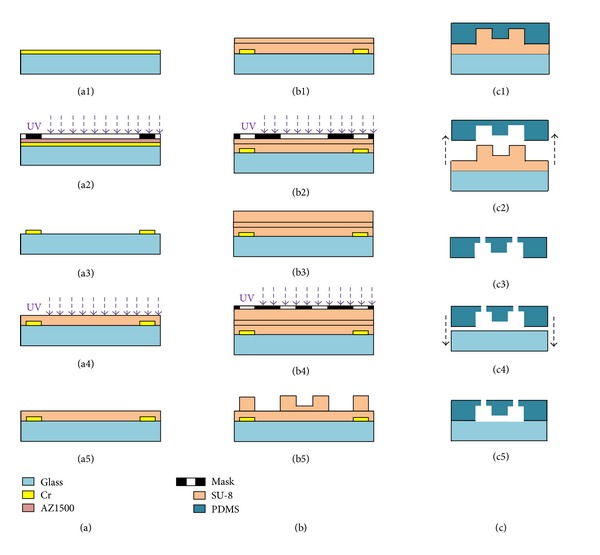
Fabrication process of the microfluidic device including Cr deposition and patterning as alignment marks and SU-8 seed layer fabrication (a), two-layer SU-8 fabrication with alignment (b), and PDMS curing and sealing with glass slides (c).

The SU-8 mold starts with glass substrate cleaning where glass slides were soaked in the glass cleaning solution (H_2_SO_4_ : K_2_Cr_2_O_7_ = 10 : 1, 8 hours) and then rinsed in DI-water (three times). A heating step on a hotplate (150°C, 30 min) was used to drive away water residuals on top of glass slides, which were then coated with Cr based on sputtering (200 nm). The deposited Cr was then spin-coated with positive photoresist of AZ1500 (1000 rpm, 1 min), prebaked (100°C, 90 sec), exposed in a mask aligner (15 mw/cm^2^, 4 sec), developed (AZ1500 developer, 50 sec), and patterned in a Cr etchant (NaOH : KMnO_4_ : H_2_O = 1 : 3 : 100, 10 min) to form alignment marks for the following two-step lithography of SU-8 (see Figure 2(a)).

For better adhesion of SU-8 structures to the patterned glass substrate, a seed layer of SU-8 was formed. More specifically, SU-8 5 was spin-coated on glass slides patterned by Cr (500 rpm, 10 sec; 2500 rpm, 35 sec), prebaked (65°C, 1 min; 95°C, 3 min), flood-exposed in the mask aligner (6 sec, 15 mw/cm^2^), postexposure-baked (65°C, 1 min; 95°C, 1 min), and hard-baked (175°C, 2 hours) (see Figure 2(a)).

Then SU-8 5 was spin-coated again to form a 5 *μ*m thick layer enabling cellular trapping, following the previously described methodology of SU-8 spin coating and prebake. Exposure at a series of exposure time (2.5 sec, 3 sec, and 3.5 sec) with mask alignment was conducted, followed by postexposure bake (65°C, 1 min; 95°C, 1 min), without development and hard bake (see Figure 2(b)). Then, the second layer of SU-8 5 was spin-coated on top of the first layer of SU-8 (500 rpm, 10 sec; 900 rpm, 35 sec), prebaked (65°C, 2 min; 95°C, 5 min), exposed with alignment (4 sec, 15 mw/cm^2^), postexposure-baked (65°C, 1 min; 95°C, 3 min), developed (SU-8 developer, 90 sec), and hard-baked (175°C, 2 hours) (see Figure 2(b)).

The PDMS molding procedure was described as follows: 75 g PDMS base and 7.5 g curing agent were mixed together at the mixing ratio of 10 : 1 and poured onto molds in a Petri dish (10 cm × 10 cm). The degassing step was then performed, followed by the curing process in an oven (80°C, 6 hours). The cured PDMS layer was then peeled away from the SU-8 mold and the through holes were punched by a hollow needle of 2 mm in diameter. Plasma oxidation (30 w, 2 min) was used to activate the surfaces of PDMS and glass slides, which were then gently put together on top of the hotplate for PDMS-glass sealing (120°C, overnight) (Figure 2(c)).

### 2.4. Device Operation

In experiments, the microfluidic devices were filled with culture medium to remove air bubbles properly. A cell suspension solution (1 million cells per mL) was injected into the main channel while the cellular trapping process was monitored by an inverted microscope (IX 71, Olympus China).

In these experiments, A549 cells were tested in experiments and their internal size distributions were used to address the effect of cellular diameters on the cellular trapping efficiency. As to the choice of pressure sources for cell injection, syringe pumping (PHD 2000, Harvard Apparatus) and gravity based pumping were used and compared, representing fluid flow at higher and lower flow rates, respectively.

## 3. Results and Discussion

Microfluidics based hydrodynamic single-cell trapping has been used to quantify cellular responses at the single-cell level, which can be classified into three stages from the perspective of technical development. In stage I, Di Carlo et al. trapped single cells based on large-array U-shape trapping weirs [20, 21] and Shoji et al. trapped single cells based on flow rate differences between two fluid channels [26, 27]. In these reported mechanisms, the trapped single cells have negligible effects on other trapping positions, and thus these trapping spots are taken randomly without a specific order. This issue leads to low trapping efficiencies in the large-array single-cell positioning [25].

To address this issue, Takeuchi et al. proposed the sequential trapping concept where the trapping sites were taken by single cells in sequence [22, 23]. In this design, for each trapping site, a bypass channel was designed to divert upcoming single cells after cellular trapping. The trapping efficiency was optimized by Lutolf et al. where the key parameter *Q*_trap_/*Q*_mc_ was optimized to values much higher than 1.00 [25]. Although a trapping efficiency higher than 97% was claimed by Lutolf et al., the proposed structure cannot realize high-density single-cell positioning.

In the third stage, Lu et al. [24] and Lee et al. [28, 29] modified the design of Takeuchi and realized high-density single-cell positioning where multiple trapping sites were placed in a sequential order together with one bypassing channel. Although a trapping efficiency of 95% was claimed, this study has a much lower *Q*_trap_/*Q*_mc_ (no higher than 0.5) and thus whether these trapping sites can be taken by cells in a sequential order is questionable, which needs further optimization.

To address this issue, in this study, we proposed a microfluidic structure including two parallel channels interconnected with cell trapping sites. By choosing inlet/outlet status, the design can be used to mimic previous designs. If the outlets of the main channel and the buffer channel are left open, the device functions as a duplicate of Shoji's design while if the outlets of the main channel and the buffer channel are left closed, the device works as a single-column counterpart of the design proposed by Lu et al.

### 3.1. Device Fabrication Characterization

The proposed SU-8 mold master contained two layers, which are 5 *μ*m and 15 *μ*m, respectively. The exact thickness was realized by regulating the spin rate of SU-8 5. After a careful parameter screening, the spin rates of 2500 rpm and 900 rpm were chosen in this study with characterized thickness of fabricated SU-8 layers shown in Table 2. It is interesting to note that the thickness of the SU-8 layer in corner areas was slightly higher than the values in the middle areas.

**Figure 3:**
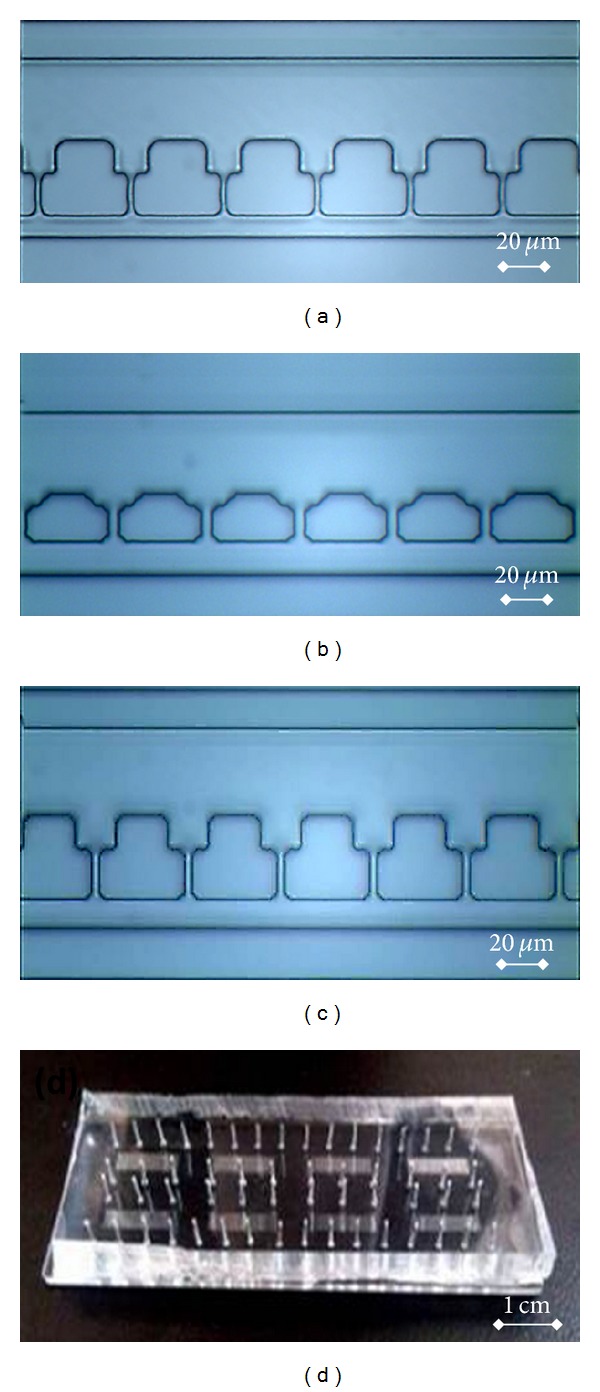
Microfluidic device fabrication results. (a) At an exposure time of 2.5 sec, channel distortion was located as an indicator of underexposure. (b) At an exposure time of 3.5 sec, channel enlargement was noticed as a sign of overexposure. (c) Exposure time of 3.0 sec produced nice device features as the optimal exposure time. (d) A fabricated prototype device.

Besides channel thickness optimization, exposure time was fine-tuned to fabricate the cell trapping channel with the height of 5 *μ*m. In the exposure time of 2.5 sec, channel distortion was located as a sign of underexposure (see Figure 3(a)) while, for the exposure time of 3.5 sec, overexposure was noticed by the channel width enlargement (see Figure 3(b)). Thus, exposure time of 3.0 sec was chosen as the optimized parameter for further device fabrication (see Figure 3(c)).  Figure 3(d) shows the fabricated prototype device for single-cell trapping.

### 3.2. Numerical Simulation Results

**Figure 4:**
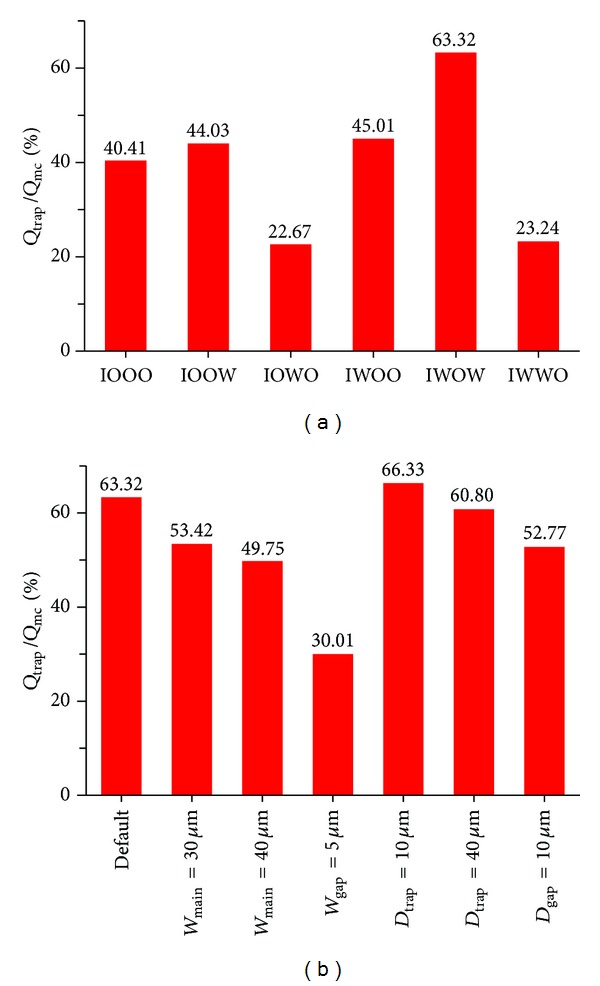
(a) *Q*_trap_/*Q*_mc_ obtained from numerical simulations as a function of channel inlet and outlet status combination as a sequence of ABCD (default geometry parameters are *W*_gap⁡_ = 10 *μ*m, *W*_main_ = 20 *μ*m, *D*_gap⁡_ = 5 *μ*m, and *D*_trap_ = 20 *μ*m, the first cellular trapping site). Note that “I” represents the channel inlet with a defined fluid flow rate, “O” represents the channel outlet with a zero pressure, and “*W*” represents the channel outlet with a zero flow rate. (b) *Q*_trap_/*Q*_mc_ as a function of geometry parameters for IWOW, showing that *W*_gap⁡_ is the key parameter and it has the most significant effect on *Q*_trap_/*Q*_mc_ among all the four parameters.

The *Q*_trap_/*Q*_mc_ of the first cellular trapping site for six inlet/outlet combinations was shown in Figure 4(a) where geometry parameters were listed as follows: *W*_main_ = 20 *μ*m, *D*_gap⁡_ = 5 *μ*m, *D*_trap_ = 20 *μ*m, and *W*_gap⁡_ = 10 *μ*m. For the case of IWOW, the setup was consistent with the device structure described in Lu's design and the *Q*_trap_/*Q*_mc_ was the highest among all the combinations (63.32%), indicating the optimal trapping efficiency. It is worth noting that since this value is much lower than 1.00, the sequential trapping of single cells along the trapping sites cannot be guaranteed.

Three setups including IOOW, IWOO, and IOOO produced comparable *Q*_trap_/*Q*_mc_ of roughly 40% and two setups of IOWO and IWWO lead to the lowest values in *Q*_trap_/*Q*_mc_ (roughly 20%). These results indicate that, for the first cellular trapping site, the status of the inlet buffer channel has a key role in determining *Q*_trap_/*Q*_mc_. As the inlet of the buffer channel was changed from the status of open “O” to wall “W”, a significant decrease in *Q*_trap_/*Q*_mc_ was recorded. Furthermore, the status of the outlets of the main channel and the buffer channel has negligible effects on *Q*_trap_/*Q*_mc_. It is worth noting that, for the case of IOOO, the channel setup was comparable to the design proposed by Shoji et al. and it has a lower *Q*_trap_/*Q*_mc_ compared to the design proposed by Lu et al.

The effect of channel geometry parameters on *Q*_trap_/*Q*_mc_ was also investigated based on numerical simulations for the case of IWOW (see Figure 4(b)). Among four parameters, *W*_gap⁡_ was shown to play a key role. As it was decreased from 10 *μ*m to 5 *μ*m, *Q*_trap_/*Q*_mc_ was decreased from 63.32% to 30.01%. For other parameters including *W*_main_, *D*_trap_, and *D*_gap⁡_, the effect on *Q*_trap_/*Q*_mc_ is less significant. More specifically, the increase in *W*_main_ from 20 *μ*m to 40 *μ*m leads to a decrease in *Q*_trap_/*Q*_mc_ from 63.32% to 49.75% and the increase in *D*_trap_ from 10 *μ*m to 40 *μ*m resulted in a decrease in *Q*_trap_/*Q*_mc_ from 66.33% to 60.80%.

### 3.3. Cellular Trapping Results

**Figure 5:**
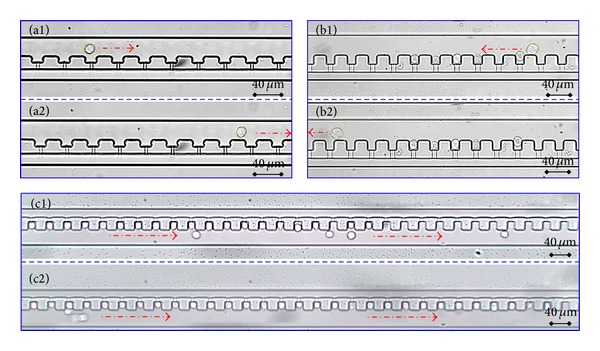
Experimental results of cellular trapping. (a) A cell under gravity was noticed to travel in the main channel without being trapped in cellular trapping spots, indicating that cellular loading cannot be realized based on gravity due to the low flow rate. (b) A coming larger cell travelled along the main channel without taking trapping sites while smaller cells were trapped in the trapping positions. These results indicated that the trapping device is sensitive to cell sizes. (c) After optimization, almost all the trapping sites were taken by individual cells. Note that pictures labelled as 1 and 2 ((a1) versus (a2), (b1) versus (b2), and (c1) versus (c2)) represent two images of the same location taken in a time sequence manner.

Figure 5 summarizes single-cell trapping results in case of IWOW with detailed geometry parameters as *W*_gap⁡_ = 10 *μ*m, *W*_main_ = 20 *μ*m, *D*_trap_ = 20 *μ*m, and *D*_gap⁡_ = 5 *μ*m. Figure 5(a) shows the cellular trapping images without proper cellular positioning when gravity was used to drive cells into the main channel. These results show that when gravity was used as the driving force to push cells into the channel at a relatively low flow rate, no cellular trapping was noticed. At relatively high flow rates based on syringe pumps, cellular trapping was noticed (see Figure 5(c)). These results indicate that, in case of single-cell trapping, the fluid flow velocity is a key parameter and it was speculated that, at low flow velocities, the fluid drag forces exerted on cells were quite limited, which may not be capable of driving cells into the cellular trapping sites.

Figure 5(b) shows the effect of cellular sizes on the trapping efficiency where a larger cell was noticed to travel along the main channel without being trapped in the trapping spots. This result suggests that the cellular trapping spots are size selective and the proposed device has difficulties in trapping larger cells. After parameter optimization, trapped single cells were shown in Figure 5(c), which indicates that although this microfluidic structure was capable of single-cell trapping, it has quite strict requirement on trapping parameters. Note that, in this cellular trapping process, trapping spots were not taken in sequence which may result from the fact that *Q*_trap_/*Q*_mc_ was much lower than 1.00.

## 4. Conclusion

In this study, both numerical simulations and experimental results were conducted to investigate the effect of capture site geometries, cellular flow velocities, and sizes on the cellular trapping efficiency. Numerical simulations confirmed that the design proposed by Lu et al. was capable of producing a higher *Q*_trap_/*Q*_mc_ than the design proposed by Shoji et al. Experimental results suggested that higher flow rates were capable of capturing single cells more easily than the lower flow rate counterpart. After fine-tuning the relevant parameters, the cellular trapping efficiency higher than 90% was realized, which may further push the development of single-cell positioning.

## Figures and Tables

**Table 1:** Key geometry parameters with chosen values used in numerical simulations. The default values are as follows: *W*_main_ = 20 *μ*m, *D*_gap⁡_ = 5 *μ*m, *D*_trap_ = 20 *μ*m, and *W*_gap⁡_ = 10 *μ*m, which were chosen based on estimated diameters of biological cells (~15 *μ*m). The height of the main channel is 20 *μ*m and the height of the cellular trapping site is 5 *μ*m.

*W*_main_	*D*_gap⁡_	*D*_trap_	*W*_gap⁡_
**20**/30/40 *μ*m	**5**/10 *μ*m	**20**/40/10 *μ*m	**10**/5 *μ*m

**Table 2:** Thickness quantification results of single-layer SU-8. Device 1 to Device 3 with expected 5 *μ*m SU-8 and Device 4 to Device 6 with expected 15 *μ*m SU-8. “C” represents the corner area and “M” represents the central area of the devices, respectively. Average ± standard variation.

(*μ*m)	C1	M1	M2	C2	Ave. ± std.
Device 1	5.0	4.7	4.7	5.1	4.88 ± 0.04
Device 2	5.1	4.6	4.6	5.1	4.85 ± 0.08
Device 3	5.0	4.6	4.7	5.0	4.83 ± 0.04
Device 4	16.2	15.1	15.2	15.2	15.43 ± 0.27
Device 5	14.4	14.5	15.4	16.4	15.18 ± 0.87
Device 6	15.5	15.3	15.0	17.5	15.83 ± 1.29
